# The mediating role of physical exercise in the relationship between university students’ interpersonal disturbance and conflict resolution strategies

**DOI:** 10.3389/fpsyg.2026.1844868

**Published:** 2026-06-17

**Authors:** Bingbing Zhong, Jun Yan, Qin Yang

**Affiliations:** College of Physical Education, Yangzhou University, Yangzhou, China

**Keywords:** interpersonal conflict resolution strategies, interpersonal disturbance, interpersonal relationship, physical exercise, university students

## Abstract

**Background:**

University represents a critical period for individual development and a key transition into wider society. Interpersonal conflicts arising from relational difficulties can lead to adverse outcomes, including risks to personal safety. This study examined the relationship between physical exercise and college students’ interpersonal relationships and conflict resolution strategies, and the mediating role of physical exercise in interpersonal relationships and conflict resolution strategies.

**Methods:**

A cross-sectional survey design was used with a sample of 1,737 undergraduate students. Data were collected using a demographic questionnaire, the Physical Activity Rating Scale, the Comprehensive Interpersonal Relationship Diagnostic Scale, and the Organizational Conflict Inventory. The relationships among variables were tested through Structural Equation Modeling (SEM), statistical significance of mediation effects was tested by bias-corrected confidence intervals for bootstrap samples.

**Results:**

The findings indicated that interpersonal disturbance had significant direct effects on multiple conflict resolution strategies, particularly collaboration and domination. Physical exercise was significantly associated with a range of strategies, including obliging, dominating, avoiding, and compromising (*β:* 0.07–0.08, *p* < 0.05). Bootstrap samples indicate that: (1) physical exercise on interpersonal disturbance and obliging was significant (*β* = 0.02, 95% CI: [0.005, 0.035]). (2) The indirect effect of physical exercise on interpersonal disturbance and compromising was significant (*β* = 0.02, 95% CI: [0.002, 0.037]). (3) Physical exercise exhibits a significant indirect effect between interpersonal disturbance and avoiding (*β* = 0.02, 95% CI: [0.003, 0.037]). (4) Physical exercise exhibits a significant indirect effect between interpersonal disturbance and dominating (*β* = 0.02, 95% CI: [0.005, 0.038]). These results suggest that physical exercise plays an important mediating role in the relationship between interpersonal disturbance and the selection of conflict resolution strategies.

**Conclusion:**

Physical exercise showed positive associations with interpersonal relationship quality and conflict resolution strategies. It is predicted that college students with interpersonal disturbances can indirectly affect the choice of conflict resolution strategy through physical exercise.

## 1 Introduction

The university period represents a critical stage in adolescent social development, during which individuals experience key transitions, including increased autonomy in decision-making, changes in academic demands, and more frequent social interactions. At the same time, university students often interact with peers from diverse regional and cultural backgrounds. Unfamiliar social contexts may heighten stress and increase the likelihood of interpersonal conflict, potentially leading to negative emotional outcomes and other adverse consequences (Bolger and DeLongis, 1989; Lee, 2008). Conflict resolution strategies reflect an individual’s level of socialisation and social skill (Courtain and Glowacz, 2019), playing a vital role in maintaining adolescent friendships (Thayer et al., 2008). Recent studies indicate that interpersonal conflict and coping strategies play an important role in university students’ mental health and social adaptation (Kandola et al., 2019; Rodriguez-Ayllon et al., 2019). However, most existing studies focus on general populations or single variables, and there is still limited understanding of the factors and mechanisms underlying conflict resolution strategies among university students. In particular, the combined roles of interpersonal disturbance and physical exercise in shaping these strategies remain unclear.

Conflict resolution strategies refer to the ways individuals handle interpersonal conflicts (Kim-Jo et al., 2010). Researchers typically classify conflict resolution strategies using the dual-concern model (Pruitt and Carnevale, 1993), which distinguishes five types—integrating, obliging, dominating, avoiding, and compromising—based on the degree of concern for one’s own and others’ interests. From a functional perspective, integrating and compromising strategies are generally associated with more adaptive interpersonal outcomes, whereas dominating and avoiding strategies tend to be associated with relationship deterioration (Putnam and Wilson, 1982). Cross-cultural studies have shown significant differences in individuals’ preferred conflict resolution strategies across cultural contexts, with those from collectivist cultures more likely to adopt cooperative and compromising approaches to maintain relational harmony (Morris et al., 1998). However, despite these classifications, the psychological and behavioral mechanisms underlying individual differences in conflict resolution strategies among university students remain insufficiently understood.

Interpersonal Disturbance (ID) refers to the psychological state of conflict or tension formed during interpersonal interactions (Chen et al., 2019). Individuals experiencing interpersonal disturbance may tend to focus on negative information during social interactions and to interpret positive social events negatively. Recent studies have further indicated that individuals with higher interpersonal disturbance are more likely to exhibit rejection sensitivity and negative cognitive biases, which in turn affect their social information processing (Iovoli et al., 2025; Preti et al., 2018). Greater interpersonal disturbance may lead individuals to reduce their use of adaptive emotional regulation strategies (Zhang and Yan, 2013), causing them to be driven by emotions when facing interpersonal conflicts. This results in an incomplete assessment of the conflict situation, ultimately leading to misguided strategies. On the other hand, prolonged interpersonal disturbances can trap individuals in a negative psychological state of social avoidance (Asher et al., 2001; Gellner et al., 2021), even leading them to evade interpersonal interactions (Ball and Gunaydin, 2022; Laursen et al., 2001). Nevertheless, most studies have concentrated on the effects of interpersonal distress on mental health, with limited attention to how it influences specific social behaviors, including conflict resolution strategies.

Beyond individual-level factors, behavioral factors such as physical exercise are also regarded as important external contributors to conflict resolution processes. As a positive lifestyle behavior, physical exercise is widely recognized for its beneficial role in promoting both physical and mental health (Biddle et al., 2019; Tomporowski and Pesce, 2019). Recent international studies have increasingly highlighted the role of physical exercise in social functioning and interpersonal behavior, showing that physical exercise is associated not only with better mental health but also with higher-quality social interactions and conflict coping styles (Bailey et al., 2013; Kandola et al., 2019; Rodriguez-Ayllon et al., 2019). From a socialization perspective, physical exercise provides structured opportunities for interpersonal interaction, thereby enhancing social connectedness and adaptability (Lin et al., 2024; Schaefer et al., 2011). Within collectivist cultures, the inherently cooperative and interactive features of physical exercise may further enhance individuals’ sense of relational awareness and social connectedness (Diener and Suh, 2003; Morris et al., 1998). In addition, evidence suggests that physical exercise improves executive functions such as inhibitory control and self-regulation (Schöndube et al., 2017; Tao and He, 2019), which may help individuals regulate emotional impulses during conflicts and adopt more constructive strategies (Murphy and Eisenberg, 1997; Tangney, 2004). However, its specific role in shaping conflict resolution strategies remains underexplored.

Social Cognitive Theory (SCT) (Bandura, 1986) provides a useful framework for understanding these relationships, proposing that behavior is shaped by the dynamic interaction of personal factors, environmental influences, and behavioral patterns. Within this framework, interpersonal disturbance and physical exercise may interactively influence individual social behavior. Empirical evidence suggests that physical exercise is negatively associated with interpersonal disturbance (Liu and Sun, 2023), while lower levels of physical exercise are associated with higher levels of defiance and distrust (Kim and Ahn, 2021). Conversely, interpersonal disturbance may also reduce participation in physical exercise due to social withdrawal and reduced peer engagement (Cao et al., 2023; Zhang and Dong, 2017). These findings suggest a complex and potentially bidirectional relationship between interpersonal disturbance and physical exercise. However, the role of physical exercise in linking interpersonal disturbance and conflict resolution strategies has not been fully examined.

Existing studies have provided a useful foundation in this area, yet several issues still require further clarification. First, empirical evidence focusing on university students remains limited, particularly in East Asian cultural contexts. Second, most research has examined the relationships among interpersonal disturbance, physical exercise, and conflict resolution strategies separately, and an integrated understanding of how these variables interact within a unified framework is still lacking. Finally, although interpersonal disturbance and physical exercise have each been linked to social functioning in previous studies, relatively few studies have considered their combined influence on conflict resolution strategies. Therefore, the present study focuses on Chinese university students and grounded in Social Cognitive Theory, investigates the relationship between interpersonal disturbance and conflict resolution strategies, with particular attention to the role of physical exercise. This study aims to clarify the psychological and behavioral mechanisms underlying conflict resolution processes and to provide theoretical and practical insights for improving interpersonal adaptation and mental health among university students.

## 2 Methodology

### 2.1 Participants

Using a convenience sampling method, questionnaires were randomly distributed across six universities in China, for a total of 1,737. After collecting the questionnaires, valid responses were screened based on the following three criteria: (1) Missing responses exceeding 30% of all items; (2) Incomplete information regarding gender, major, or only-child status; (3) Questionnaires with identical selections across all items in the scale. After excluding invalid questionnaires based on the above criteria, 1,334 valid questionnaires were collected, yielding a response rate of 76.82%.

Of the participants, 693 were male (51.95%) and 641 were female (48.05%). In addition, 942 participants (70.61%) were single-child families. Participants ranged in age from 17 to 22 years, with a mean age of 19.45 ± 1.25 years. The sample size was determined using Cochran’s formula (Cochran, 1977):

$$n=\frac{z^{2}\times p(1-p)}{d^{2}}$$

The sample size is determined using the statistical formula. “*z”* denotes the *z*-score corresponding to the confidence level, typically set at 2.58 to achieve 99% confidence. *p* denotes the expected proportion of a specific characteristic within the target population, often set at 0.50 to maximize sample size when preliminary data is unavailable; *d* indicates the acceptable precision, usually set at 0.05. Based on these parameters, the minimum sample size required for this study is 666. With the required level of precision, 600 to 700 samples can achieve a 99% confidence level and an error rate of no more than 5%. Therefore, the 1,737 questionnaires distributed in this study far exceeded the required minimum sample size, ensuring the reliability and representativeness of the statistical results. In addition, all participants provided written informed consent prior to participation.

### 2.2 Data collection tools

All scales used in this study have been validated among Chinese college students and show good reliability and validity.

#### 2.2.1 Physical exercise status

The Physical Activity Rating Scale (PARS-3), revised by Liang (1994), was used to assess individuals’ physical exercise habits. Although the original scale is named the Physical Activity Rating Scale, it has been widely applied in Chinese research to measure physical exercise behavior. This scale assesses exercise volume based on three dimensions: intensity, duration, and frequency of physical exercise. Exercise volume = intensity × duration × frequency. Intensity and frequency range from 1 to 5 levels, scoring 1–5 points, respectively. Duration is rated on a 1–5 scale, with 0–4 points. The maximum score is 100 points, and the minimum is 0 points. Exercise volume assessment criteria: ≤19 points indicates low exercise volume; 20–42 points indicates moderate exercise volume; ≥43 points indicates high exercise volume. In this study, the Cronbach’s *α* coefficient for the scale was 0.735.

#### 2.2.2 Interpersonal relationships

The Interpersonal Relationship Integrative Diagnostic Scale, developed by Zheng (1999) was adopted. This scale diagnoses and measures interpersonal relationships across four dimensions: socializing, conversational skills, interpersonal conduct, and interactions with the opposite sex. This scale consists of 28 items, each answered with either “Yes” or “No.” Specifically, a total score ranging from 0 to 8 indicates good interpersonal relationships. A score between 9 and 14 suggests mild interpersonal disturbance. A score between 15 and 28 indicates significant interpersonal disturbance. Higher total scores reflect greater levels of interpersonal disturbance. In this study, the Cronbach’s α coefficient for the scale was 0.899. The Cronbach’s α coefficients for the subscales of interpersonal conversation, interpersonal friendship, social interaction, and opposite-sex relationships were 0.735, 0.788, 0.640, and 0.716, respectively. A confirmatory factor analysis was conducted using the maximum likelihood (ML) method for parameter estimation. Model fit was evaluated based on commonly accepted criteria: RMSEA values below 0.08 indicate an acceptable fit, and values below 0.05 indicate a good fit; CFI, TLI, GFI, IFI, and NFI values above 0.90 indicate an acceptable fit (Hu and Bentler, 1999; Kline, 2018). The results of confirmatory factor analysis were X^2^/df = 4.45, CFI = 0.903, GFI = 0.914, TLI = 0.887, IFI = 0.904, NFI = 0.879, RMSEA = 0.051, and SRMR = 0.057. These findings indicate that the model achieved an acceptable level of fit.

#### 2.2.3 Interpersonal conflict resolution strategies

This study employed the Revised Organizational Conflict Inventory (ROCI-II) Student Version developed by Zhang and Liu (2010) to examine university students’ use of conflict resolution strategies. The revised Conflict Resolution Strategies Questionnaire for Students comprises four dimensions—obliging, dominating, avoiding, and compromising—with a total of 28 items scored on a 5-point scale. Higher scores on each dimension indicate a greater tendency for individuals to employ the corresponding strategy in conflict situations. In this study, the Cronbach’s *α* coefficient for the total scale score was 0.957. The Cronbach’s α coefficients for the obliging, compromising, avoiding, and dominating subscales were 0.958, 0.900, 0.863, and 0.783, respectively. Confirmatory factor analysis was conducted using the maximum likelihood (ML) method for parameter estimation, and the model fit criteria were the same as those reported in Section 2.2.2. The confirmatory factor analysis results were X^2^/df = 6.98, CFI = 0.936, GFI = 0.880, TLI = 0.924, IFI = 0.936, NFI = 0.926, RMSEA = 0.067, and SRMR = 0.075. These findings indicate that the model achieved an acceptable level of fit.

### 2.3 Statistical analysis

Data processing and preliminary analyses were conducted using SPSS 26.0. Before running the structural equation model, the data were pre-processed and examined through missing data handling, descriptive statistics, and correlation analysis. Independent-samples t-tests were also conducted to compare gender differences across variables, providing a basis for subsequent analyses.

Based on this, structural equation modeling (SEM) was conducted using AMOS. Model parameters were estimated using the maximum likelihood (ML) method, which is appropriate for continuous variables and performs well with large samples. The significance of mediation effects was tested using a bias-corrected bootstrap approach with 5,000 resamples to generate 95% confidence intervals. Effects were considered statistically significant when the 95% bootstrap confidence interval did not include zero. Standardized estimates were reported for all structural paths.

## 3 Research findings

### 3.1 Common method bias test

Since all research variables were measured using self-report methods, common method variance may be present. Harman’s common method variance test was employed. Principal component analysis was employed to evaluate the unrotated factor analysis results. The first factor explained 24.00% of the variance, which is below the 40% critical threshold. Therefore, no significant common method bias was present.

### 3.2 Descriptive statistics and correlation analysis

The mean values, standard deviations, and correlation coefficients for the variables among the subjects in this study are presented in Table 1.

**Table 1:** Descriptive statistics and correlation analysis of major variables (*n* = 1,334).

Variable/dimension	1	2	3	4	5	6
1. Physical exercise	–					
2. Interpersonal Disturbance	0.22^**^	–				
3. Obliging	0.10^**^	0.09^**^	–			
4. Compromising	0.11^**^	0.13^**^	0.78^**^	–		
5. Avoiding	0.09^**^	0.09^**^	0.85^**^	0.91^**^	–	
6. Dominating	0.12^**^	0.13^**^	0.84^**^	0.86^**^	0.88^**^	–

*p < 0.05, **p < 0.01.

The results indicate that significant correlations exist among all major variables. Interpersonal disturbance showed a positive correlation with physical exercise (*r* = 0.22, *p* < 0.01) and with various conflict resolution strategies (*r* = 0.09–0.13, *p* < 0.01). Physical exercise showed positive correlations with all conflict resolution strategies, with *r* values ranging from 0.09 to 0.12 (*p* < 0.01). Positive correlations were also found between obliging, dominating, avoiding, and compromising strategies, with *r* values ranging from 0.80 to 0.91 (*p* < 0.01). Participants reported moderately severe interpersonal disturbance.

Additionally, results from the independent samples *t*-test indicate that males reported higher levels of physical exercise compared to females (*p* < 0.01) and employed compromising and dominating strategies less frequently (*p* < 0.05) (Table 2).

**Table 2:** Analysis of gender differences among college students in research variables and their dimensions.

Variable/dimension	Gender		*t*	Cohen’s d
	Male (*n* = 693)	Female (*n* = 641)		
Physical exercise	22.32	10.57	12.03**	0.66
Interpersonal Disturbance	22.30	21.96	1.14	0.62
Obliging	37.11	36.78	0.85	0.05
Compromising	21.99	22.66	−2.91**	−0.16
Avoiding	21.52	21.78	−1.22	−0.07
Dominating	17.56	17.98	−2.38*	−0.13

*p < 0.05, **p < 0.01.

### 3.3 The influence of interpersonal disturbance on interpersonal conflict resolution strategies: the mediating role of physical exercise

**Figure 1:**
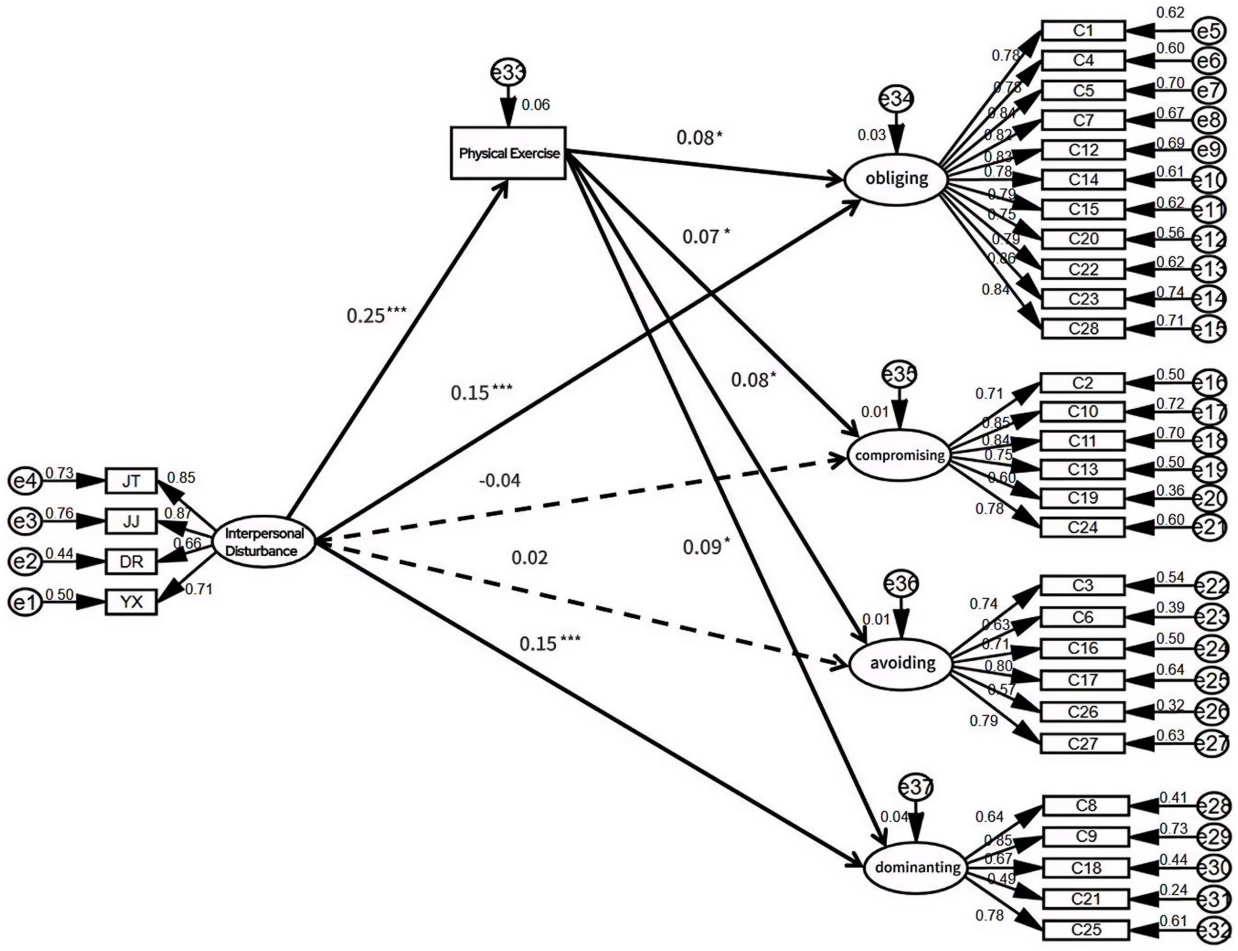
Path model of interpersonal disturbance and physical exercise on conflict resolution strategies.

Using Amos to construct a structural equation model, we examined the indirect effects of physical exercise on interpersonal disturbance and various conflict resolution strategies. Standardized path coefficients (*β*) are reported for all structural relationships. The results indicate that the model fits well: RMSEA = 0.065, CFI = 0.919, TLI = 0.908, NFI = 0.906, and X^2^/df = 6.66. Interpersonal disturbance significantly and directly influenced obliging (*β* = 0.15) and dominating (*β* = 0.15) (*p* < 0.001), while its direct effects on compromising (*β* = −0.04) and avoiding (*β* = 0.02) were insignificant (*p* > 0.05). The path from interpersonal disturbance to physical exercise was significant (*β* = 0.25, *p* < 0.001). Physical exercise significantly influenced obliging, dominating, compromising, and avoiding (*β* = 0.07–0.09, *p* < 0.05) (Figure 1).

All indirect effects were tested using a bias-corrected bootstrap procedure with 5,000 resamples, as shown in Table 3. Physical exercise significantly mediated the relationship between interpersonal disturbance and obliging (*β* = 0.02, 95% CI: [0.005, 0.035]), compromising (*β* = 0.02, 95% CI: [0.002, 0.037]), avoiding (*β* = 0.02, 95% CI: [0.003, 0.037]), and dominating (*β* = 0.02, 95% CI: [0.005, 0.038]).

**Table 3:** Path coefficients of the mediation model and test of mediation effects.

Path	*β*	95% CI		*p*	Proportion of relative effect
		*LLCI*	*ULCI*		
Interpersonal disturbance → physical exercise → obliging	0.02	0.005	0.035	0.008	5.32%
Interpersonal disturbance→ physical exercise → compromising	0.02	0.002	0.037	0.024	5.04%
Interpersonal disturbance → physical exercise → avoiding	0.02	0.003	0.037	0.026	5.32%
Interpersonal disturbance → physical exercise → dominating	0.02	0.005	0.038	0.009	5.88%
Total indirect effect	0.08	0.025	0.138	0.005	21.85%
Total effect	0.36	0.089	0.622	0.009	100%

## 4 Discussion

### 4.1 Patterns and gender dimorphism in conflict resolution strategies

University students, among the groups most prone to interpersonal issues, find that their problem-solving approaches are crucial for maintaining relationships and safeguarding their mental health. This study aims to explore the influence of interpersonal disturbance among university students on conflict resolution strategies and the mechanisms underlying these strategies. The findings reveal that university students prefer obliging problem-solving strategies, employ the least non-adaptive dominating strategies, and use compromising and avoiding strategies at intermediate levels compared to other strategies. Overall, university students employed positive coping strategies more frequently than negative ones, consistent with previous research findings (Chen and Sun, 2024). From the perspective of SCT, such behavioral tendencies can be understood as the result of dynamic interactions among personal factors, environmental influences, and behavioral reinforcement. Furthermore, the findings indicate significant gender differences in university students’ use of compliance and dominating strategies, with females exhibiting a greater tendency than males to employ these strategies when confronting conflict situations—a pattern partially consistent with previous research (Zou, 2007). One possible reason is that women are generally more inclined to express their emotions and to pay greater attention to others’ feelings and needs. When faced with conflict, they may be more willing to compromise or yield to avoid hurting others’ feelings or damaging interpersonal relationships.

### 4.2 The mediating role of physical exercise in managing interpersonal disturbance

The results of the structural equation model indicate that physical exercise significantly mediates the relationship between interpersonal disturbance and various conflict resolution strategies. Firstly, interpersonal disturbance may increase physical exercise, a finding inconsistent with previous studies (Liu and Sun, 2023; Zhang and Dong, 2017). Based on the SCT, individuals’ behavioral choices under interpersonal disturbance are influenced not only by negative emotional states but also by self-regulatory processes and environmental reinforcement. Physical exercise, as a behavior that provides immediate emotional regulation and opportunities for social interaction, may serve as an important coping strategy for managing interpersonal disturbance. Previous studies have largely suggested that interpersonal disturbance can lead individuals to experience negative emotions, perceive physical exercise as a psychological burden, and develop aversion toward it. In fact, physical exercise is an effective means of stress relief, helping to alleviate stress-related symptoms such as fatigue, anxiety, perceived stress, and depression (Jonsdottir et al., 2010). Moreover, university students may be more likely to choose exercise over sleep or eating as a way to manage stress (Vogel et al., 2022). On the other hand, physical exercise provides a platform for group interaction for those struggling with interpersonal relationships. To satisfy their need for group belonging, they may engage more in physical exercise (Chen et al., 2017), seeking to connect with others and gain recognition. Additionally, physical exercise increases the use of relatively positive strategies such as obliging and compromising, partially consistent with existing research findings that exercise is significantly positively correlated with social problem-solving abilities (Sone et al., 2017). Physical exercise is not only linked to physical health but also closely associated with an individual’s mental well-being and social behavior. It enhances self-control abilities (Schöndube et al., 2017; Zhong, 2024), reduces hostility and impulsivity (Li and Zhang, 2023; Tangney, 2004), and makes individuals more inclined to respond to challenges and conflicts in social interactions with a calm attitude. Additionally, physical exercise enhances self-awareness while fostering concern for others and empathy, aiding in understanding others’ perspectives and in attending to their needs (Lee et al., 2002). This may encourage individuals to adopt more positive strategies, such as obliging, for conflict resolution.

### 4.3 Competitive contexts in physical exercise and maladaptive conflict resolution

The findings also indicate that physical exercise is significantly positively correlated with the use of maladaptive strategies such as dominating and avoiding. From a SCT perspective, behavioral patterns are shaped not only by individual experience but also by environmental contexts and reinforcement processes. A possible reason is that some sports emphasise competition rather than cooperation. Previous studies have shown that obliging and competition in both electronic and real-world social games may influence physiological and psychological changes. Competitive games tend to increase aggression, while cooperative games enhance motivation and promote prosocial behavior (Marker and Staiano, 2015). Therefore, it can be argued that prolonged participation in competitive sports may also foster an individual’s habitual pursuit of victory or attempts to dominate others, making them more inclined to adopt dominant and competitive strategies in conflicts. Additionally, the selection of conflict resolution strategies is influenced by the specific context of the conflict and individual characteristics (Zhang, 2002). Research indicates that individuals high in neuroticism and high in concealment tendencies are less likely to employ adaptive strategies (Wang, 2005). Those high in agreeableness and extraversion are more inclined to choose strategies that balance both parties’ interests (Johnson and Ostendorf, 1993; Park and Antonioni, 2007), and when addressing conflicts arising from misunderstandings, they tend to favors confrontational strategies (Zhang, 2002).

In summary, physical exercise plays a significant role in mediating interpersonal disturbance and in the use of conflict resolution strategies. University students experiencing interpersonal disturbance can indirectly influence their choice of conflict resolution strategies through physical exercise.

## 5 Conclusion

Physical exercise is positively correlated with interpersonal relationships and conflict resolution strategies. It positively predicts that university students experiencing interpersonal disturbance may indirectly influence their choice of conflict resolution strategies through physical exercise.

## 6 Practical implications

Resolving interpersonal conflicts holds significant importance for the social development of university students. Not only does it benefit their physical and mental well-being, but it also helps them adapt more smoothly to the social environment during their socialization process. This study elucidates the mechanism by which individuals experiencing high interpersonal disturbance tend to alleviate psychological pressure by increasing physical exercise. In conflict situations, they are more likely to adopt adaptive conflict resolution strategies in a rational, proactive manner. On the other hand, numerous contextual and individual characteristics play a significant role in selecting conflict resolution strategies. This study providing empirical support for the development of intervention strategies to prevent interpersonal conflicts and improve interpersonal relationships.

## 7 Limitations

The present study has several limitations that should be acknowledged. First, the cross-sectional design limits causal inference; future research could employ longitudinal or experimental approaches to address this issue. Second, the data were primarily derived from self-report questionnaires, which may be influenced by social desirability and subjective bias; future studies could incorporate behavioral measures or multi-source data to improve objectivity. Third, the mediating effect of physical exercise was confirmed in this study, but the variability of different types of physical exercise could be further refined in future studies by examining exercise type, intensity, and participation motivation.

## Data Availability

The original contributions presented in the study are included in the article/supplementary material, further inquiries can be directed to the corresponding authors.
